# A novel approach for haplotype-based association analysis using family data

**DOI:** 10.1186/1471-2105-11-S1-S45

**Published:** 2010-01-18

**Authors:** Yixuan Chen, Xin Li, Jing Li

**Affiliations:** 1 Electrical Engineering and Computer Science Department, Case Western Reserve University, Cleveland, OH 44106, USA

## Abstract

**Background:**

Haplotype-based approaches have been extensively studied for case-control association mapping in recent years. It has been shown that haplotype methods can provide more consistent results comparing to single-locus based approaches, especially in cases where causal variants are not typed. Improved power has been observed by clustering similar or rare haplotypes into groups to reduce the degrees of freedom of association tests. For family-based association studies, one commonly used strategy is *Transmission Disequilibrium Tests *(TDT), which examine the imbalanced transmission of alleles/haplotypes to affected and normal children. Many extensions have been developed to deal with general pedigrees and continuous traits.

**Results:**

In this paper, we propose a new haplotype-based association method for family data that is different from the TDT framework. Our approach (termed F_HapMiner) is based on our previous successful experiences on haplotype inference from pedigree data and haplotype-based association mapping. It first infers diplotype pairs of each individual in each pedigree assuming no recombination within a family. A phenotype score is then defined for each founder haplotype. Finally, F_HapMiner applies a clustering algorithm on those founder haplotypes based on their similarities and identifies haplotype clusters that show significant associations with diseases/traits. We have performed extensive simulations based on realistic assumptions to evaluate the effectiveness of the proposed approach by considering different factors such as allele frequency, linkage disequilibrium (LD) structure, disease model and sample size. Comparisons with single-locus and haplotype-based TDT methods demonstrate that our approach consistently outperforms the TDT-based approaches regardless of disease models, local LD structures or allele/haplotype frequencies.

**Conclusion:**

We present a novel haplotype-based association approach using family data. Experiment results demonstrate that it achieves significantly higher power than TDT-based approaches.

## Background

Identification and localization of disease susceptibility genes is an important step towards the understanding of etiology of diseases and the development of new approaches for diagnoses and treatments. With the aid of molecular markers, statistical methodologies have made fundamental contributions to the identification of a substantial number of Mendelian diseases. However, deciphering genetic architectures of complex diseases is still a great challenge. With the advance of technology in recent years, single-nucleotide polymorphisms (SNPs) have emerged as the primary molecular marker for genetic mapping. SNPs are suitable for unbiased genome-wide assessments as well as fine-scale mapping because they provide a (nearly) complete coverage over the whole genome with high density. However, great challenges exist in analyzing hundreds of thousands of SNPs from thousands of individuals, not only because of the high volume and high dimensionality of data, but also because of their complicated interrelated structure, known as haplotypes.

Driven by the international HapMap project [1], considerable information about haplotype structures and haplotype frequencies among several populations has been obtained. Haplotype-based association mapping approaches, which take into consideration of correlated SNP structures, have drawn much interests and many new methodologies have been developed [2-8]. (For the discussion of the possible advantages of haplotype-based approaches over single SNP based approaches, see [8] and references therein.) In particular, methodologies that explicitly examine haplotype sharing patterns from case-control samples using various clustering algorithms have shown initial success [4,6-8], all of which are based on the assumption that haplotypes from cases are expected to be more similar than haplotypes from controls in regions near the disease genes.

In an earlier work, our group has proposed an algorithmic approach and developed a program called HapMiner, for haplotype mapping of disease genes utilizing a density-based clustering algorithm [8]. HapMiner is based on the assumption that, the haplotype segments with recent disease mutations, tend to be close to each other due to linkage disequilibrium, while other haplotypes can be regarded as random noises sampled from the haplotype space. The algorithm takes haplotype segments as data points in a high dimensional space. Clusters are then identified based on a similarity measure using the density-based clustering algorithm. Significance of association of each cluster is then evaluated. It has been shown that HapMiner can effectively obtain meaningful information from noisy datasets because of the concept of "density-based" clusters. More recently, we have extended HapMiner to quantitative trait mapping based on haplotype information from unrelated individuals [9]. Haplotype uncertainties can also be taken into consideration [10].

Almost all haplotype-based methods mentioned above including HapMiner use the case-control design, and most of them require haplotype/diplotype information which must be inferred from genotype data. However, the case-control design for association studies may suffer from population stratification [11] and haplotype inference from un-related individuals may contain uncertainties [12]. On the other hand, association approaches based on family data (such as TDT and their variants [13,14]) are robust against population admixture, and haplotype inference using family data normally achieves much more reliable results [12]. Several TDT-type tests using haplotype information have been proposed (*e.g.*, [15-17]). Recently, Qian [18] adopted the haplotype sharing correlation (HSC) method to detect phenotype and haplotype associations based on family data. The author has shown that the HSC method achieved higher power than single- and multi-locus based methods. However, the HSC method requires phased haplotype data as input and does not work if no recombination presents within a pedigree. Given the high densities of existing SNP chips and moderate family sizes in practice, even for large number (hundreds, even thousands) of SNPs, recombination events within a family are extreme rare. In this paper, we combine our previous work on haplotype inference from family data and haplotype-based association into one unified framework. The approach first infers haplotype configurations for each pedigree assuming no recombination using our most recent haplotyping algorithm [19]. A phenotype score is then defined for each founder haplotype. Assuming all founder haplotypes are independent, the HapMiner algorithm is then applied. We compare the approach, termed F_HapMiner, with the single-locus and haplotype-based TDT methods implemented in two popular programs [17,20] under a variety of disease models and penetrance values with realistic haplotype frequencies and local LD structures. Experiment results show that our approach consistently achieves higher power than TDT-based approaches.

## Methods

**Figure 1:**
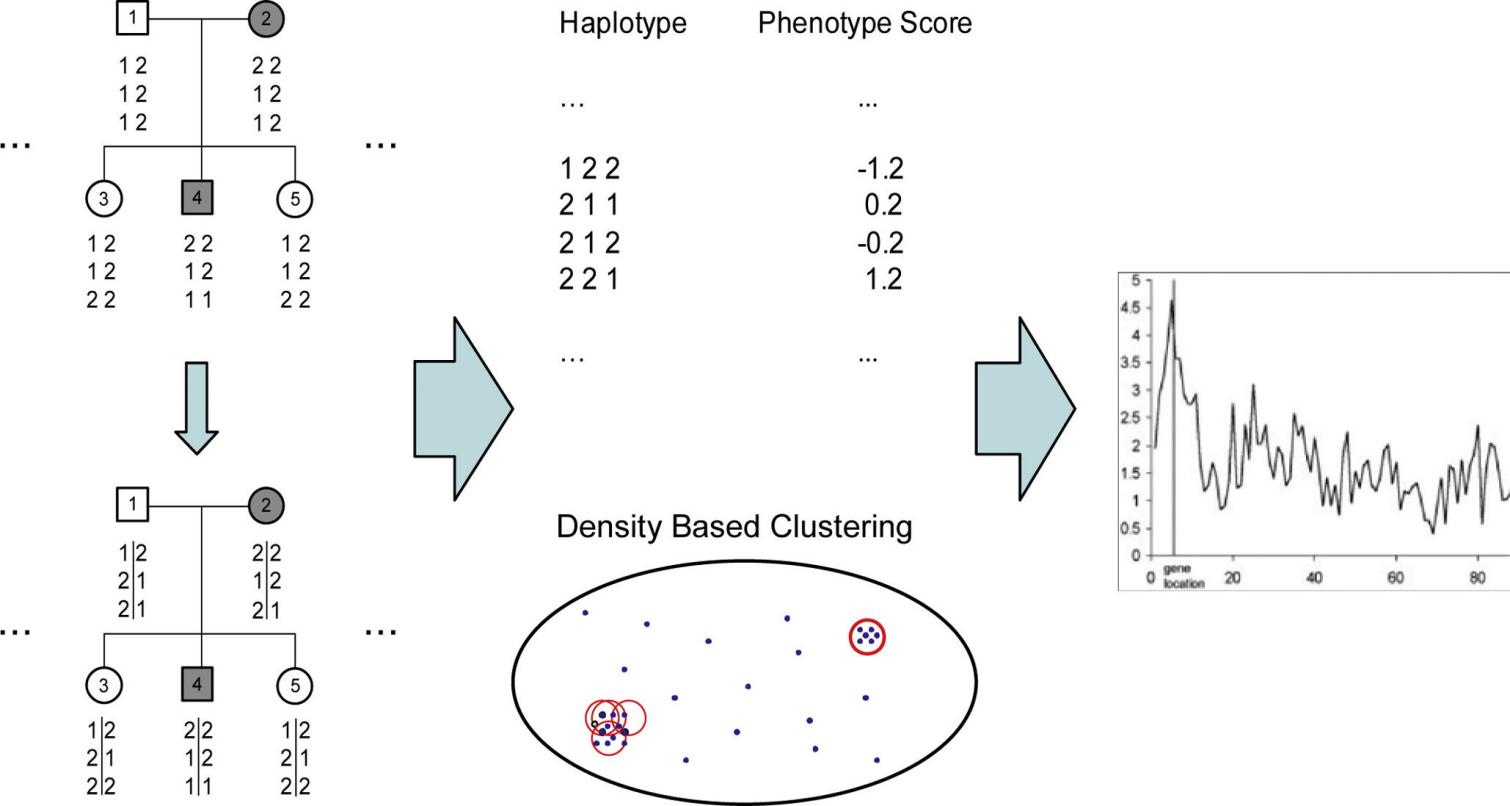
**The computational framework of the proposed approach F_HapMiner**. (1) Infer haplotypes on all families based on the DSS algorithm. (2) Calculate the phenotype score for each founder haplotype from each family based on its occurrences in affected and normal members. (3) Cluster all the founder haplotypes using a position weighted haplotype similarity measure. (4) Evaluate the correlation between clusters and the trait using a statistical test.

Our primary focus is on candidate gene studies using highly linked markers (*e.g.*, SNPs). Candidate regions might be obtained from previous linkage analysis or some other prior knowledge. We assume no recombination events within each pedigree in a candidate region. For each region, family-based approaches (*e.g.*, [18]) in general cannot distinguish one marker from the other because there are no recombination events. However, by combining family and population information, F_HapMiner can define a statistical score for each marker, indicating the strength of its association with the trait. The approach consists of two major steps. Firstly, pedigree data are used to infer haplotype configurations for each pedigree [19]. And a phenotype score is defined for each founder haplotype in a family based on the phenotypes of family members who possess that haplotype. (Thus identical founder haplotypes from different families may have different phenotype scores.) Secondly, all founder haplotypes from all families, which can be assumed unrelated, together with their associated phenotype scores will be evaluated at the population level using the HapMiner algorithm [9]. Our proposed approach can deal with families with arbitrary sizes. Both continuous and binary traits can be considered. Haplotype configurations will be inferred using our most recent haplotype inference algorithm named PedPhase.DSS [19]. Founder haplotypes are then clustered using a position weighted similarity measure [8] and each cluster is evaluated by comparing its phenotypic mean with the overall phenotypic mean [9]. Our approach is different from TDT-type procedures, because essentially haplotype-based TDT tests treat each haplotype as a distinct allele, while our approach considers haplotype similarities. The overall procedure is summarized in Figure 1 with details in sequel.

### Haplotype inference

**Figure 2:**
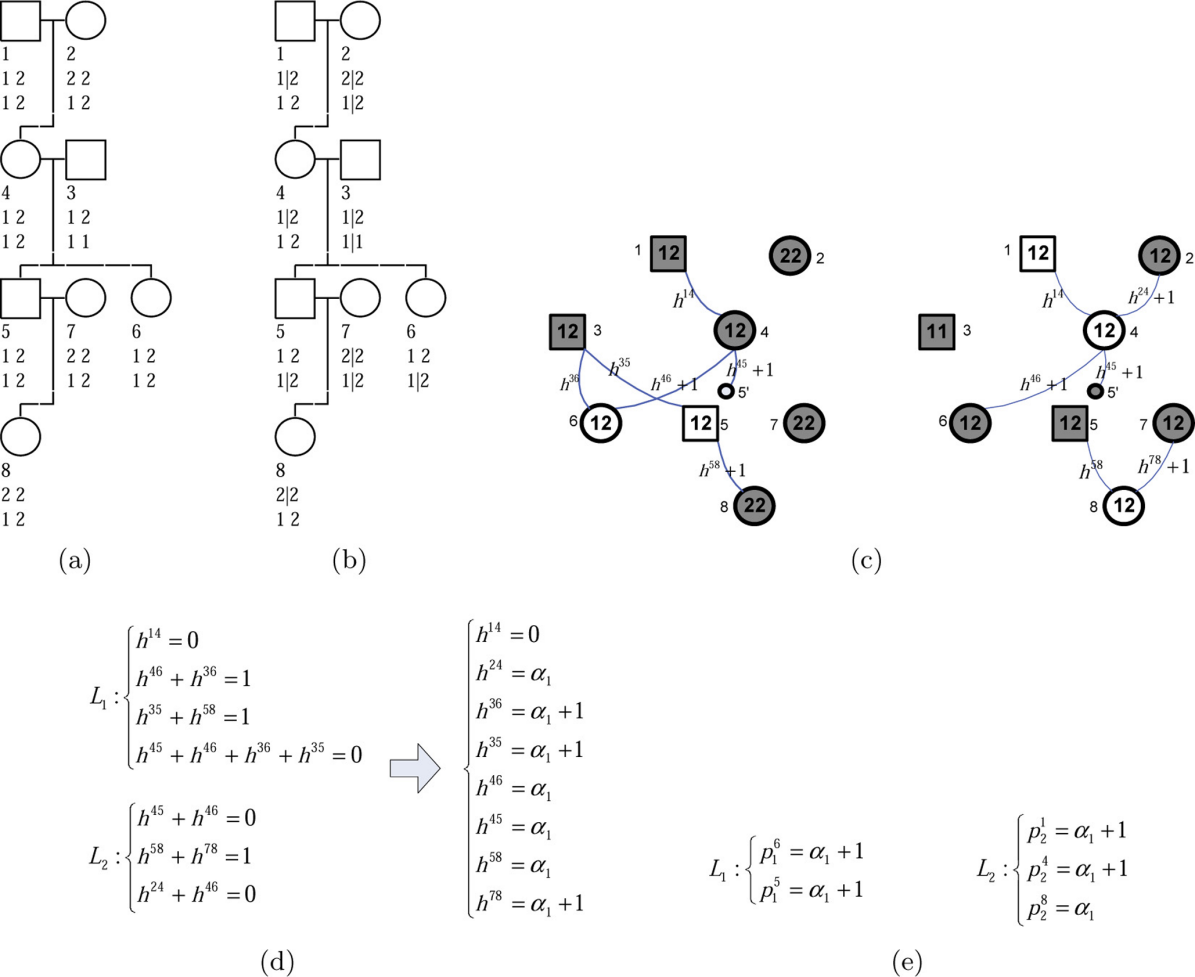
**Illustration of the DSS algorithm**. a) The input pedigree with 8 members and their genotype data. b) Haplotypes are partially determined based on the Mendelian law and denoted as (paternal | maternal). In addition, one heterozygous SNP is fixed in each founder (an individual without parents in the pedigree). Five SNPs are left with freedom. c) Locus graphs for the two loci. Each graph has the same set of nodes as the original pedigree, where shaded (predetermined) nodes representing fixed SNPs in b). Each child is linked to its heterozygous parents, with edges labeled using *h*-variables. Node 5 is duplicated for easy process. d) Left: constraints on *h*-variables collected based on the two locus graphs using disjoint-set structures. Constraints are collected based on each pair of linked predetermined nodes (or duplicated nodes), and only no redundant ones are kept. Right: Solutions of *h*-variables can be obtained directly based on the disjoint-set structures. They are represented by free variables *α*. In this example, only one degree of freedom. e) Solutions of *p *variables derived from *h *variables in d).

Haplotype inference in general is hard, even with family information. The total number of consistent haplotype assignments can be very large depending on the size of the pedigree and missing patterns. When there are no recombinations within a family and no missing alleles, efficient optimal algorithms do exist [19,21]. One of our most recent developments based on disjoint-set structures (denoted as the DSS algorithm [19]), can effectively handle family data with no recombinants. For the special case of data with no missing, it is an (almost) linear time algorithm for pedigrees with no loops. Briefly, the algorithm first formulates genotype constraints as a linear system of inheritance variables. It then utilizes disjoint-set structures to encode connectivity information among individuals, to detect constraints from genotypes, and to check consistency of constraints. Here we use an example (Figure 2) to illustrate the basic idea of the algorithm and details can be found in [19]. Figure 2.a shows the input genotypes of a pedigree with eight members for 2 SNPs. After one applies the Mendelian law (the paternal allele is separated from the maternal one by a "|") and fixes one heterozygous locus in each founder (members 1, 2, 3, 7), there are 5 heterozygous loci left to be determined (Figure 2.b). But not all of them are free due to the constraint of no recombination. Those uncertainties about heterozygous SNPs are represented by *p *variables. The DSS algorithm first constructs locus graphs, one for each SNP (Figure 2.c), which connect each child with their heterozygous parents through inheritance (*h*) variables. The 0-recombination constraint is enforced by using a single *h *variable for each parent-child pair throughout all SNPs. The algorithm then traverses these locus graphs to collect constraints, to check the consistency/conflict and to obtain solutions of *h *variables and then *p *variables using disjoint-set structures. At the end, there is only one degree of freedom in *h *variables (Figure 2.d). Once that freedom is fixed, all *p *variables are also fixed (Figure 2.e).

### Phenotype score for founder haplotypes

The general principle in defining a phenotypic value for each founder haplotype is to ensure that haplotypes occurring more often in affected/high risk members within a pedigree will receive higher values. There are different ways to define phenotype scores for each founder haplotype and different definitions may have different effects on subsequent analyses. Let *Y*_*ij *_denote the phenotype of the *j*^*th *^member in the *i*^*th *^family and *J*_*i *_is the size of pedigree *i*. Let *h*_*ik *_denote the *k*^*th *^founder haplotype in the *i*^*th *^family and let *s*_*ik *_denote its score to be defined. Let *H*_*ij *_denote the set of the haplotype pair of the *j*^*th *^member in the *i*^*th *^family. One simple measure that resembles the relative risk concept but limited to a single pedigree can be defined as:(1)

where *I*(*x*) is the indicator function and *t *is a user defined threshold. Alternatively, one can define(2)

as suggested by Qian [18], where  = Σ_*j*_*Y*_*ij*_/*J*_*i *_is the average of the trait values in family *i*. The phenotype value *Y*_*ij *_can be the quantitative trait value itself or 1/0 for affected/normal individuals. We have tested both measures and preliminary results show that the latter measure has slightly better results. Therefore, we report only results using the second measure in our experiments.

### Mining founder haplotypes

The founder haplotypes are treated as independent. Together with their phenotypic scores, they serve as the input of the HapMiner program [8,9]. The basic assumption of HapMiner is that due to linkage disequilibrium, disease-associated haplotypes are expected to be more similar to each other than haplotypes randomly drawn from the population. Therefore, HapMiner directly explores the sharing of haplotype segments that observe extreme phenotypes. The measure of sharing between two haplotypes is defined by a position weighted similarity score, which combines the length of the shared segments and the number of identical alleles around a given marker position. For each marker position, a haplotype segment centered at the position will be clustered based on the similarity measure. Each cluster is evaluated using a *Q*-score, which is defined based on the deviation of the phenotypic mean of the cluster from the mean of all samples (*t*-statistic). The highest score among all clusters is taken as the score of that marker position. The significant level can be obtained via a permutation test (However Bonferroni correction for multiple testing is used in the simulation for efficiency). More details about the algorithm can be found in [8,9].

### Simulations

We evaluate the performance of F_HapMiner using extensive simulations with realistic parameters. The simulation consists of three steps. We first obtain population haplotype frequencies from two datasets, representing different marker densities and haplotype/genotype frequencies. The first dataset is based on the Cystic Fibrosis (CF) study [22] and the second one is the simulated dataset from the Genetic Analysis Workshop (GAW) 15 [23]. CF data is a well-known dataset that has been examined by many researchers. We take the same 29 haplotypes and their frequencies estimated by Becker and Knapp [24] (also see Table A1 in Additional file 1). The total length of the region is 1.8 Mb with 19 loci, but marker interval distances vary dramatically. The second dataset is a portion of the simulated data from the GAW 15, which was used to model the complex genetic architecture of rheumatoid arthritis (RA). We randomly choose 500 families from the first replicate, and take a segment of 20 SNPs from chromosome 6 centered at the HLA-DRB1 locus. The average marker interval distance is about 10 kbp. The haplotypes of each individual are known and their frequencies are estimated based on their counts in parents. The total number of distinct haplotypes is 65 (Table A2 in Additional file 1). For both datasets, only haplotypes and their frequency distributions (Tables A1 & A2 in Additional file 1) are used in our simulation. We refer data generated based on these frequencies as CF and GAW dataset, respectively. To generate a set of realistic pedigrees in step two, we directly sample family structures from the 65 pedigrees of the CEPH study [25]. A family in this dataset may have two (13 out of 65) or three (52 out of 65) generations with 4-20 members (average 13). Figure A1 in Additional file 1 shows one typical CEPH family structure. Given a set of parameters, we generate pedigrees one by one as follows. First, one CEPH pedigree is randomly selected. Second, for the pedigree structure selected, each founder will be assigned two haplotypes, sampled independently based on the population haplotype distribution defined in step one. Haplotypes of non-founders are obtained based on Mendelian law assuming no recombination. In the third step, we assign phenotypes to each member in each family based on two different disease models: a single-locus model and a rare haplotype model. For the single locus model, we assume only one SNP in the region will increase the risk of being affected. The penetrance, which defines the probability of being affected given a specific genotype at the risk locus, will take realistic values. The disease status of each individual will be determined based on the genotype at the risk locus. To evaluate the effect of disease allele frequencies, we take each SNP in turn as the risk SNP, which will then be removed before applying any statistical methods. Therefore, statistical power will mainly depend on local LD strength and structures. In addition, we also consider a rare haplotype model, *i.e.*, a few rare haplotypes may increase the risk of being affected. This model is to simulate some common complex diseases that might have haplotype effect or allele heterogeneity, or simply common diseases caused by multiple rare mutations [26]. For the haplotypes obtained from the CF dataset, there are 22 rare haplotypes with the same frequency (0.01786). For the haplotypes obtained from GAW 15, a haplotype is regarded as a rare haplotype if its population frequency is less than 0.02. A certain number of (2 to 6) haplotypes from these rare haplotypes are randomly selected as risk haplotypes with the same effect. Individuals carrying one or two disease haplotypes will have higher risks to be affected. The effect of each risk haplotype is also defined based on penetrance. Only pedigrees with at least one affected member will be retained. More pedigrees with genotypes/haplotypes and phenotypes can be generated in the same way, until a specified number of pedigrees (which is a parameter) can be reached.

## Results

### Parameters

We investigate the performance of F_HapMiner by considering different population parameters, and in comparison with two variants of TDT-based methods. Some important factors that affect the statistical power of any approach include linkage disequilibrium and haplotype patterns within the region, risk allele/haplotype frequency, disease penetrance, and sample size. We model LD/haplotype patterns by directly sampling founder haplotypes from haplotype distributions of real data (CF) and simulated data (GAW 15). In terms of risk allele frequencies, each SNP has been taken as the risk locus once for the single-locus model, with minor allele frequencies range from 0.00045 to 0.482. For the rare haplotype model, 2-6 haplotypes with small frequencies are randomly chosen as risk haplotypes. Penetrance, which represents the effect size of a disease locus, is another important parameter. One can specify an ordered triple (*p*_*A*|*dd*_, *p*_*A*|*dD*_, *p*_*A*|*DD*_) as the penetrance set, where each element represents the penetrance of having 0, 1, or 2 disease alleles or haplotypes. We use three penetrance sets (Table 1). Set C is adopted from Qian's study [18], which happens to correspond to a population prevalence around 0.1 when the risk allele frequency is 0.1. We further choose two additional sets with smaller penetrance (A and B in Table 1), and their population prevalence ranges from 0.07 to 0.15 and 0.028 to 0.09 respectively, for allele/haplotype frequency from 0.1 to 0.5. Their relative effect sizes can also be illustrated using the concept of genotype relative risks (λ_1 _= *P *(*A*|*Dd*)/*P *(*A*|*dd*) and *λ*_2 _= *P *(*A*|*DD*)/*P *(*A*|*dd*)), as illustrated in Table 1. Table 1 also provides the distribution of different genotypes given the affected status when the disease allele frequency *p*_*D *_= 0.1. Different sample sizes (*i.e.*, number of pedigrees) are considered for power comparisons of three approaches: F_HapMiner, a single-locus TDT implemented in PLINK [20], and a haplotype-based TDT implemented in FBAT [17]. The power of an approach is defined as the percentage of detecting significant associations in 100 replicates after adjustment of multiple testing.

**Table 1:** Penetrance sets and their effects. G: genotype, d: normal allele/haplotype, D: disease allele/haplotype, A: affected.

Penetrance Set	Values	*λ*_1_	*λ*_2_	*Pr*(*G *= *dd|A*)	*Pr*(*G *= *dD|A*)	*Pr*(*G *= *DD*|*A*)
A	(0.05, 0.15, 0.25)	3	5	0.579	0.386	0.036
B	(0.01, 0.1, 0.15)	10	15	0.293	0.652	0.054
C	(0.05, 0.3, 0.5)	6	10	0.407	0.543	0.050

### Type I error

To assess the power of different approaches of detecting significant associations between SNPs and traits, it is important to have a proper control of false positive discoveries due to chance (*i.e.*, type I errors). In this study, we set the overall error rate to be 0.05 after Bonferroni correction of multiple testing for all experiments. The type I error rate of each method was estimated as the proportion of significant associations reported in all replicates under the null model in which no SNP or haplotype carries disease risks. The average false positive rates over all parameter combinations tested for F_HapMiner (with the haplotype segment length of 1), the single-locus TDT and the haplotype-based TDT are 1.1%, 3.6%, and 6.3% respectively. Single-locus TDT is slightly conservative and F_HapMiner is quite conservative. We suspect that the primary reason for this is due to Bonferroni correction of multiple testing. On the other hand, no correction is needed for the haplotype-based TDT and it tends to have a slightly inflated type I error rate.

### Power

We performed extensive experiments to evaluate the proposed approach. The results were organized into two subsections based on different disease models (*i.e.*, single SNP *vs*. rare haplotype model). For each model, we first evaluated the effect of the haplotype segment length parameter of F_HapMiner and chose a proper length for each of the models for remaining tests. For the single-locus disease model, we have examined the relationship of LD structure and mapping precision. We then compared the power of our approach and the single-locus TDT approach using different penetrance values and examined the effect of allele frequencies. For the rare haplotype model, we have investigated the power of the three approaches using different penetrance values, different number of rare haplotypes and different sample sizes.

#### Single locus model

**Figure 3:**
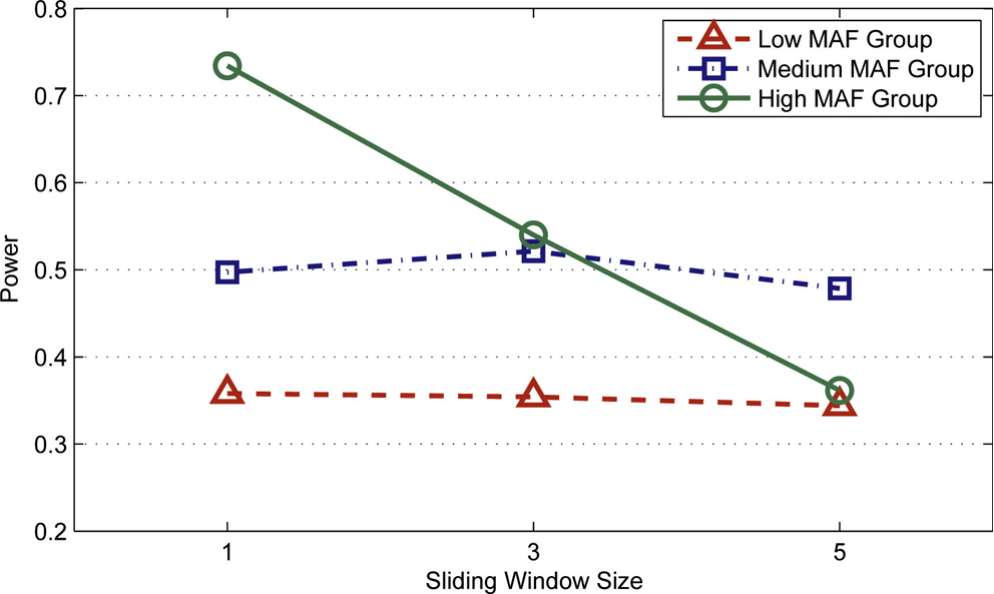
**The effect of sliding window sizes**. The power of F_HapMiner on the single locus model using different sliding window sizes, grouped according to MAF (Table 2). The power may be adversely affected with large window sizes when haplotype blocks are short (the case of SNPs in the group with high MAF). Result is based on 50 pedigrees on the CF dataset using the penetrance set A.

**Figure 4:**
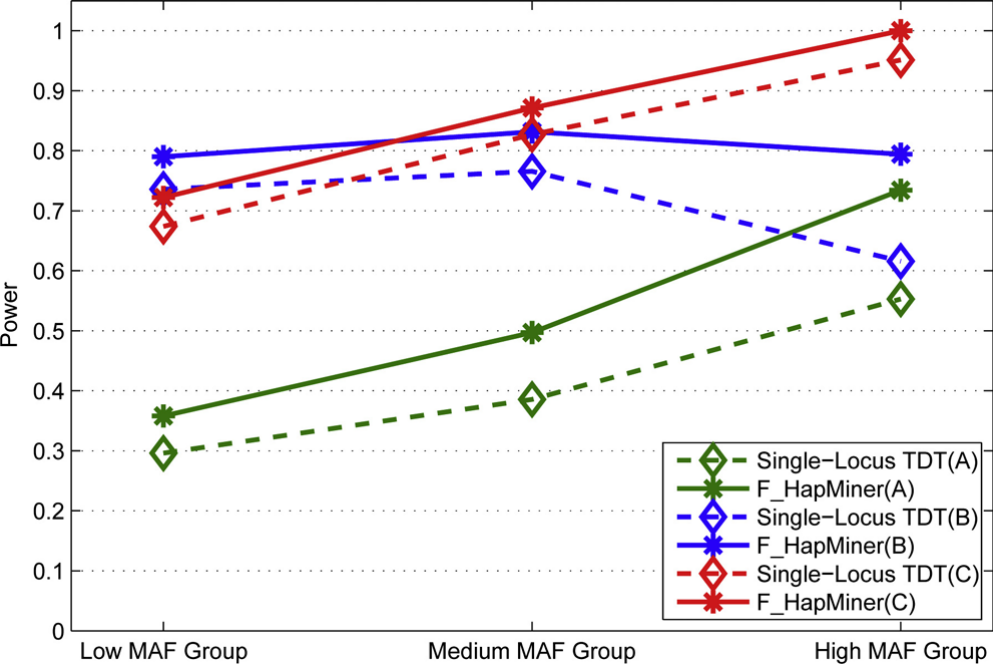
**Power comparison on the single-locus model**. Power of F_HapMiner and the single-locus TDT on the CF dataset using three different penetrance models. Result is based on 50 pedigrees and grouped according to MAF. The letter inside the parentheses indicates the penetrance set.

When evaluating the association of phenotypes and haplotypes, F_HapMiner uses a sliding window approach with an important parameter of the haplotype segment length. The motivation of using haplotype segment is to capture untyped risk SNPs or haplotype effect. Ideally one should use a variable window size to closely capture the variation of local LD structure (which is currently under consideration). In the existing implementation of F_HapMiner, users have to use a fixed window size. We first tested the effect of this parameter on both datasets. Results indicated that the precise behaviors critically depend on LD structures. Figure 3 shows the results of different segment lengths using the CF dataset for the penetrance set A with a sample size of 50 pedigrees. For easy illustrations, SNPs were separated into three groups based on their minor allele frequencies. The information about minor allele frequencies, group memberships, as well as power for each individual SNPs (for the segment length of 1, *i.e.*, a single SNP) can be found in Table 2. Based on Figure 3, one might conclude that with high MAF, the power of F_HapMiner deteriorates significantly when one increases the segment length, while it shows no or only slight decreases with low to medium MAF. However, by carefully examining the LD structure of the region (Figure A2 in Additional file 1), we believe that the real reason behind this result is that the SNPs with high MAF (SNPs 1, 3, 5, 16, 17, 18, and 19) happen to be in short haplotype blocks. Therefore, extending haplotype segments beyond block boundaries actually brought noises to the analysis. Results based on GAW 15 dataset also showed the consistent trend. Therefore, for the single locus disease model, we chose the window size of 1 for F_HapMiner and only compared its performance with the single-locus TDT approach. The results are shown in Table 2 and Figure 4. In almost all cases (Table 2), F_HapMiner constantly outperforms the single-locus TDT, regardless of penetrance models, allele frequencies, or LD structures. The improvement is more significant when the power of the single-locus TDT is in the range of 35% to 85%. We believe that the gain of F_HapMiner is mainly from two sources. First, we inferred the inheritances and haplotypes by considering all SNPs jointly while TDT only took into accounts of individual informative markers. Second, the phenotype scores defined earlier indeed captured the correlations between the disease and the risk SNP. In terms of disease effects (the three penetrance sets), both approaches have worst performance on Set A, reflecting its weak signals. Allele frequencies also greatly impact the results. Both approaches have very low power for the two SNPs with extremely low MAF (≤ 5.5%, first two rows in Table 2). On average, the power increases with the increase of allele frequencies for penetrance Sets A & C (Figure 4). However, LD patterns can greatly affect results, even for SNPs with similar allele frequencies. For example, SNP 4 has a MAF of 0.21. But the power is very low for both approaches and for all three penetrance models. LD analysis illustrates that SNP 4 has very low LD with other SNPs (Figure A2 in Additional file 1). For the penetrance set B, the power (*vs. *allele frequencies) of both approaches behaves differently: the average of power actually declined when allele frequencies increased (the Medium MAF group *vs. *the High MAF group). Furthermore, both approaches were more powerful on the low MAF group and less powerful on the high MAF group for the set B, comparing to the results on the set C. These differences actually reflect the fact that approaches behave differently for different models with respect to allele frequencies (many previous papers have shown that in the extreme case of recessive models versus dominant models, approaches behave differently when allele frequencies vary). Our results demonstrate that detection of genetic variants responsible to diseases is tricky even for a single risk SNP if it is not typed. The success (power) depends on many interrelated factors including disease models, allele frequencies and LD patterns. Results (Table A3 in Additional file 1) on the GAW dataset also support our observations.

**Table 2:** The power of F_HapMiner and the single-locus TDT (SL-TDT) on each SNP for different penetrance models using the CF dataset (sample size = 50 pedigrees). SNP are ordered and grouped based on their minor allele frequencies (MAF).

			Penetrance Set A		Penetrance Set B		Penetrance Set C	
					
Group	MAF	SNP	SL-TDT	F_HapMiner	SL-TDT	F_HapMiner	SL-TDT	F_HapMiner
Low	0.03572	16	0.07	0.07	0.44	0.56	0.24	0.38
	0.05358	2	0.08	0.02	0.34	0.39	0.31	0.23
	0.08925	13	0.40	0.55	0.96	1.00	0.89	1.00
	0.10711	11	0.51	0.55	0.95	1.00	0.98	1.00
	0.12502	8	0.42	0.60	0.99	1.00	0.95	1.00
Medium	0.14283	9	0.52	0.74	0.98	1.00	0.99	1.00
	0.14283	15	0.41	0.55	0.94	1.00	0.97	1.00
	0.14287	14	0.38	0.42	0.88	0.95	0.93	0.98
	0.14288	10	0.59	0.80	0.98	1.00	1.00	1.00
	0.16074	7	0.28	0.31	0.67	0.85	0.77	0.95
	0.21425	4	0.06	0.02	0.14	0.08	0.16	0.17
	0.26789	12	0.46	0.64	0.77	0.94	0.97	1.00
High	0.35716	3	0.42	0.58	0.64	0.84	0.95	1.00
	0.41073	18	0.74	0.85	0.65	0.90	0.99	1.00
	0.42859	19	0.69	0.85	0.73	0.87	1.00	1.00
	0.4286	1	0.38	0.58	0.52	0.67	0.86	1.00
	0.46427	6	0.64	0.82	0.68	0.80	0.98	1.00
	0.48213	5	0.57	0.78	0.55	0.81	0.99	1.00
	0.48216	17	0.43	0.68	0.54	0.67	0.89	1.00

**Figure 5:**
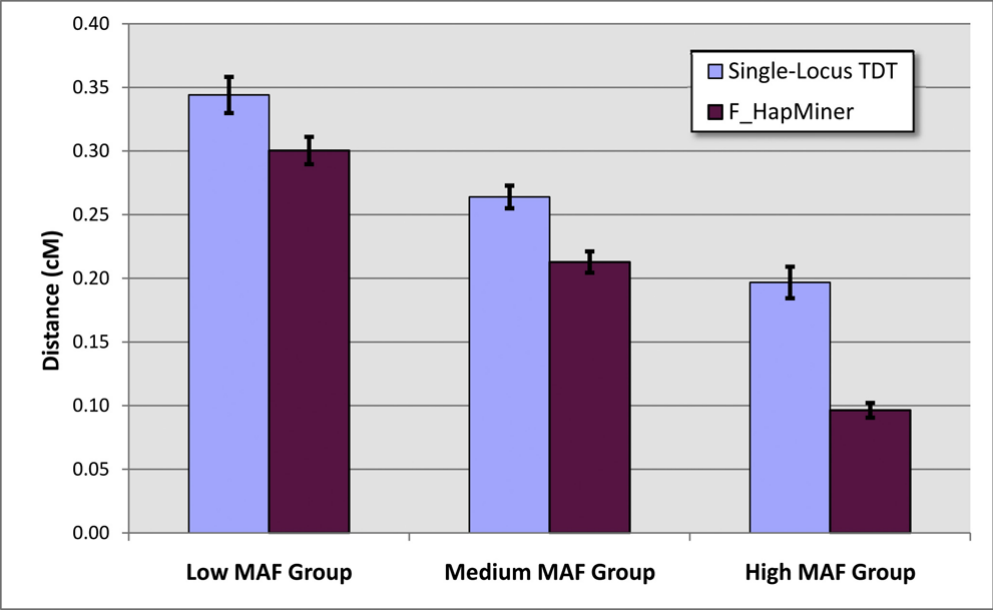
**Mapping precision on the single-locus model**. Average distances in centimorgan from the predicted SNP to the true risk SNP for F_HapMiner and the single-locus TDT using the CF data. Result is based on 50 pedigrees using using the penetrance set A and grouped according to MAF.

Both F_HapMiner and the single-locus TDT reported *p*-values for each marker position. We then examined the mapping precision of both approaches, defined as the genetic distance between the SNP with the lowest *p*-value and the real risk SNP based on the original map file of the CF study. On average, results from F_HapMiner are more accurate than those from the single-locus TDT (Figure 5), which is consistent with the results that F_HapMiner archived higher power. Overall, the prediction of F_HapMiner on penetrance set A is about 28% closer comparing to the TDT. Higher minor allele frequencies in general improve the mapping precision.

#### Rare haplotype model

In this experiment, we randomly selected 2 to 6 rare haplotypes from each dataset as risk haplotypes. For each number of risk haplotypes, we made 10 random selections from the dataset. For each selection, 100 replicates were generated. The results were grouped and averaged based on the number of risk haplotypes. We compared the performance of F_HapMiner, the single-locus TDT and the haplotype-based TDT. For F_HapMiner, we first tested the effect of the sliding window size of 5 to 10. Preliminary results (data not shown) demonstrated that F_HapMiner achieved slightly higher power for longer haplotype segment lengths, but with increased running time. By considering all these factors, as well as the block structures, we decided to use the window size of 10. Because the total frequency of all risk haplotypes is low, one would expect that it is harder to detect associations. Indeed, we had to increase the sample size to at least 100 families to ensure all methods had some power to detect signals for the penetrance set A.

**Figure 6:**
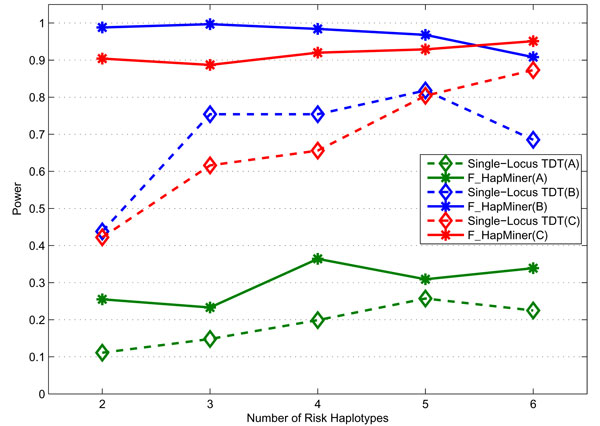
**Power comparison on the rare haplotype model**. Power of F_HapMiner and the single-locus TDT on the CF dataset using three penetrance sets, grouped based on the number of risk haplotypes. The letter inside the parentheses indicates the penetrance set. Result is based on 100 pedigrees per replicate.

**Figure 7:**
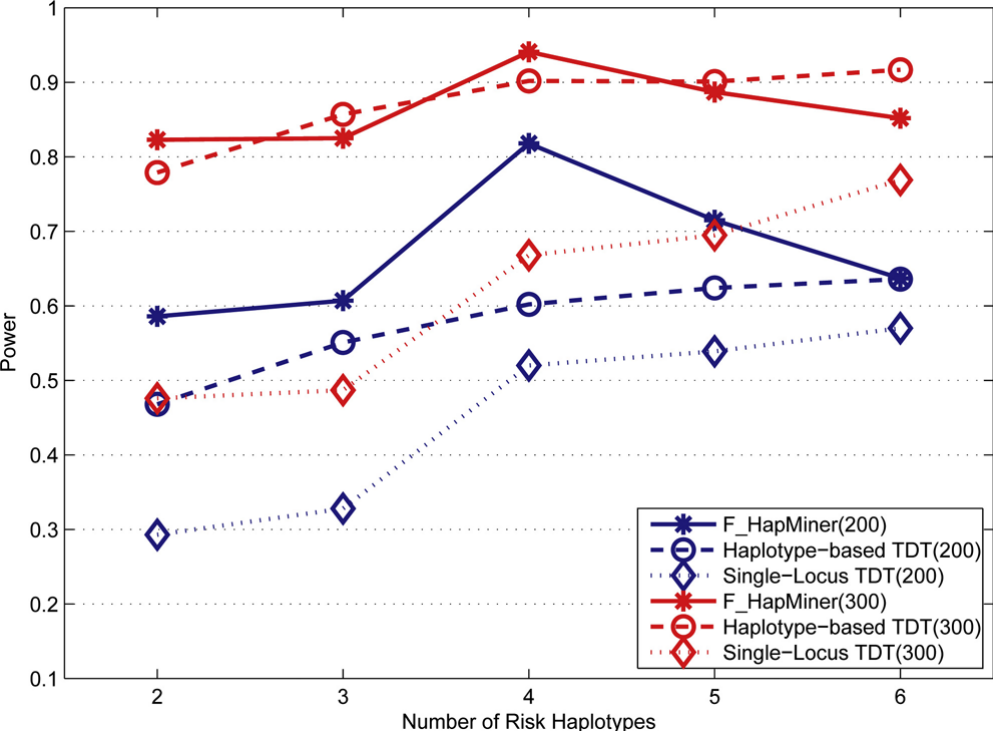
**Power comparison with the haplotype-based TDT**. Power of F_HapMiner, the single-locus TDT and the haplotype-based TDT on the CF dataset using the rare haplotype model with the penetrance set A for different sample sizes.

As expected, for the rare haplotype model, both haplotype-based approaches performed significantly better than the single-locus TDT (Figures 6 &7). In particular, for all three penetrance models, F_HapMiner outperformed the single-locus TDT by a margin of 9% to 130% using the CF dataset (Figure 6). The result certified that F_HapMiner had mostly captured the haplotype effect, comparing with the single-locus TDT, which examined each SNP one at a time. Such an advantage is more significant when the number of rare risk haplotypes is small. For example, the power of F_HapMiner has doubled with 2 risk haplotypes. Similar trend has been observed using the GAW dataset (Table 3). There are other interesting observations. One has to double the sample size when using the GAW data (Table 3, 200 families/replicate) to achieve comparable power as the CF data (Figure 6, 100 families/replicate). The primary reason is that the estimated rare haplotype frequencies in the GAW data are much lower than those from the CF data (Tables A1 & A2 in Additional file 1). All approaches have lower power with low frequencies from GAW data. Furthermore, because of the low frequencies, both F_HapMiner and the single-locus TDT performed much better using the penetrance set B comparing with the set C, which was also consistent with our earlier observation that approaches tended to perform better for the model B when allele frequencies were low (Table 2). The haplotype-based TDT also outperformed the single-locus TDT (Figure 7). For the two haplotype-based approaches, their power was comparable when using 300 pedigrees. However, F_HapMiner achieved much higher power than the haplotype-based TDT in the case of 200 pedigrees, with one exception when using 6 rare haplotypes, in which case they had the same power (Figure 7). The result demonstrates that F_HapMiner has higher sensitivity to moderate signal. Figure 7 also shows that the performance of the haplotype-based TDT gradually increased with more rare haplotypes, while the power of F_HapMiner deteriorated with high number of risk haplotypes (e.g., 5 and 6). On one hand, this seems to make sense because F_HapMiner only outputs the most significant cluster. On the other hand, the behaviors of both approaches actually depend more on the fact that which haplotypes are selected as risk haplotypes and how similar they are (Figures 6, 7 and A3 in Additional file 1), not so much on the *number *of rare haplotypes. As expected, the power of all approaches will increase with the increase of sample sizes (Figures 7 and A3 in Additional file 1). We notice that a better method might achieve higher power even with smaller number of samples (F_HapMiner with 200 families *vs. *the single-locus TDT with 300 families in Figure 7). Furthermore, the power usually does not have a linear relationship with the sample size. With the same number of increased samples, an approach will gain much more when its power is low (Figures 7 and A3 in Additional file 1).

**Table 3:** Power of F_HapMiner and the single-locus TDT (SL-TDT) using the GAW dataset on the rare haplotype model (sample size = 200 pedigrees).

	Penetrance Set A		Penetrance Set B		Penetrance Set C	
	
Risk Haplotype #	SL-TDT	F_HapMiner	SL-TDT	F_HapMiner	SL-TDT	F_HapMiner
2	0.06	0.12	0.36	0.58	0.17	0.30
3	0.08	0.07	0.54	0.73	0.27	0.42
4	0.09	0.13	0.65	0.90	0.41	0.68
5	0.11	0.17	0.60	0.92	0.63	0.88
6	0.10	0.30	0.90	0.97	0.55	0.73

## Discussion

We have proposed a new approach for family-based haplotype association testing and fine mapping. For a given candidate region, we assume that no recombination has been observed within each pedigree. The approach consists of three steps. First, we use a novel algorithm to infer diplotype pairs of each individual in each pedigree. Our previous experiments have shown that the DSS haplotype inference algorithm is very efficient and accurate when there are no recombinations and when missing genotypes are randomly distributed across SNPs and members. The DSS algorithm can also handle families with a few recombinants. In the current implementations, when there exist multiple haplotype assignments with zero recombinant, we randomly select one as the true solution. This might adversely affect the results. More recently, we extend DSS to use population information to select the most likely solution [27], which may further improve the power. However, even this new approach cannot effectively handle data with many untyped members (usually founders) that may happen in real data. The difficulty lies in the fact that in this case one cannot use the zero recombinant assumption to effectively limit the search space. We currently investigate new formulations and approaches for such cases. In the second step, a phenotype score is defined for each founder haplotype to measure its correlation with the phenotype. Haplotypes that appear more frequently in affected/high risk members tend to receive higher scores. On the contrary, haplotypes in normal members will get lower scores. This way, the haplotype-phenotype correlation embedded in descendants is collected and accumulated to founder haplotypes. Such information is then used in subsequent haplotype-based association tests using a clustering approach based on haplotype similarities in step three. Extensive experiments demonstrate that our approach outperforms the single-locus and haplotype-based TDTs, on both the single-locus disease model and the rare haplotype model. F_HapMiner has several advantages over the haplotype-based TDT approach implemented in FBAT. Our experience shows that FBAT requires large number of samples to obtain haplotype population frequencies. For instance, FBAT cannot process about 7% of the total replicates when the sample size is 200. Additionally, FBAT also has limits on the total number of different haplotypes, which implies that it cannot handle large regions with more SNPs.

We generated simulated data based on the haplotype frequency distributions of two datasets (CF and GAW 15). Our experiments show that haplotype patterns (diversity, frequencies, LD structures) have profound impacts on the power to detect associations. On the contrary, marker interval distances have less effect. Our disease models probably have higher relative risks comparing to real complex diseases. The sizes of CEPH pedigrees are much larger than many real studies. In practice, for genotype relative risks of 1.2 to 1.5, and/or for families with smaller sizes, a much large number of samples are needed. More tests on different disease models and pedigree sizes are warranted. Furthermore, a test of the approach on a real data set from our collaborators is currently underway. We also observe that the single-locus TDT also has some power in detecting associations for the rare haplotype model (Figure 6). Further detailed analysis tells us that the power actually came from different SNPs, whose frequencies happen to be close to the frequencies of rare haplotypes.

F_HapMiner can be extended in a few ways. Firstly, it relies on users to specify the sliding window size for the clustering procedure. The optimal value depends on the input data (*e.g.*, local LD structures) and the characteristics of the underlying disease. One solution is to determine the sliding window size based on the LD structure of the data. The sliding window may extend in both directions until current SNP is in low LD with next one. We are currently investigating new approaches to automatically adjust this parameter. Secondly, a phenotype score is calculated for each founder haplotype to represent its correlation with the disease. Whether a score can reflect the real correlation is crucial to the performance of F_HapMiner. Ideally, phenotype scores of disease-related haplotypes should have higher values with small variance. In the future, we will investigate new phenotype scores to further improve the power of F_HapMiner. Finally, we assume zero recombination in the candidate region and infer haplotype based on this assumption. This assumption can be relaxed as we are extending our haplotype inference algorithm to allow recombinations.

## Conclusion

In summary, we have presented a novel haplotype-based approach of association testing and fine mapping using family data. Simulation results have shown that our approach F_HapMiner outperforms the single-locus TDT for both the single locus model and the rare haplotype model. F_HapMiner also has advantages over the haplotype-based TDT when the sample size is moderate.

## Competing interests

The authors declare that they have no competing interests.

## Authors' contributions

JL initiated the project and designed the experiments. YC carried out the experiments. XL performed the haplotype inference experiment. All three together drafted the manuscript.

## Supplementary Material

Additional file 1 Click here for file
