# Supplementary material for: A novel approach for haplotype-based association analysis using family data
Source: BMC Bioinformatics. 2010 Jan 18;11(Suppl 1):S45. doi: 10.1186/1471-2105-11-S1-S45 (PMC3009518; doi:10.1186/1471-2105-11-S1-S45)

Appendix

Table A1 - Haplotype frequency distribution of CF dataset.

Serial No.	Haplotype	Frequency
1	1 1 1 2 2 2 1 1 2 1 2 1 2 1 2 1 1 1 1	0.19643
2	1 1 1 2 2 2 1 1 2 1 2 1 2 1 2 1 2 2 2	0.17857
3	2 1 2 2 2 2 1 1 2 1 2 1 2 1 2 1 1 1 1	0.07143
4	2 1 2 2 1 1 1 1 2 1 2 1 2 1 2 1 2 2 2	0.05357
5	1 1 1 1 1 1 1 1 2 1 2 1 2 1 2 1 2 1 1	0.03571
6	2 1 2 1 1 1 1 1 2 1 2 2 2 2 2 1 2 2 2	0.03571
7	2 1 2 2 1 1 1 1 2 1 2 1 2 1 2 1 1 1 1	0.03571
8	1 1 1 1 1 1 1 1 2 1 2 1 2 1 2 1 1 1 1	0.01786
9	1 1 1 1 1 1 2 2 1 2 1 2 1 1 1 1 2 2 2	0.01786
10	1 1 1 1 2 2 1 1 2 1 2 1 2 1 2 1 1 1 1	0.01786
11	1 1 1 1 2 2 1 1 2 1 2 1 2 1 2 1 2 2 2	0.01786
12	1 1 1 2 1 1 1 1 2 1 2 1 2 1 2 1 2 2 2	0.01786
13	1 1 1 2 1 1 1 2 1 2 1 2 1 1 1 2 2 1 1	0.01786
14	1 1 1 2 1 1 2 1 1 2 1 2 1 1 1 1 2 2 2	0.01786
15	1 1 1 2 1 2 1 1 2 1 2 1 2 1 2 1 1 1 1	0.01786
16	1 1 2 2 1 1 1 2 1 2 1 2 1 1 1 1 1 1 1	0.01786
17	2 1 1 1 2 2 1 2 1 2 1 2 1 1 1 1 2 1 2	0.01786
18	2 1 1 2 1 1 2 1 2 1 2 2 2 2 1 1 1 1 1	0.01786
19	2 1 2 1 1 1 1 1 2 1 2 1 2 1 2 1 1 1 1	0.01786
20	2 1 2 1 1 1 1 1 2 1 2 2 2 2 2 1 1 1 1	0.01786
21	2 1 2 2 1 1 1 2 1 2 2 1 2 1 2 2 2 2 2	0.01786
22	2 1 2 2 1 1 1 2 1 2 2 2 2 1 1 1 1 1 1	0.01786
23	2 1 2 2 1 1 2 1 2 1 2 2 2 2 2 1 1 1 1	0.01786
24	2 1 2 2 1 1 2 1 2 1 2 2 2 2 2 1 2 2 2	0.01786
25	2 1 2 2 1 1 2 2 1 2 1 2 2 1 1 1 1 1 1	0.01786
26	2 1 2 2 2 2 2 1 2 1 2 2 2 2 2 1 2 2 2	0.01786
27	2 2 1 1 1 1 2 1 2 1 2 1 2 1 2 1 1 1 1	0.01786
28	2 2 1 2 1 1 1 1 2 1 2 1 2 1 2 1 2 2 2	0.01786
29	2 2 1 2 1 1 2 1 2 1 2 2 2 2 2 1 1 1 1	0.01786

Table A2 - Haplotype frequency distribution of GAW dataset.

Serial No.	Haplotype	Frequency
1	1 1 1 1 1 1 1 1 1 1 1 1 1 1 1 1 1 1 1 1	0.432
2	2 1 2 2 1 1 1 1 1 2 2 2 2 2 1 2 1 1 1 2	0.093
3	1 1 1 1 1 1 1 1 1 1 1 1 1 1 1 1 1 1 2 1	0.0845
4	1 2 1 1 1 2 2 2 2 1 1 1 2 1 1 2 2 2 1 1	0.0485
5	2 1 2 2 1 1 1 1 1 2 2 2 2 2 1 2 1 1 2 2	0.0365
6	1 1 1 2 1 1 1 2 2 2 2 1 2 2 1 2 1 1 2 1	0.0315
7	1 1 1 2 1 1 1 1 1 2 2 2 2 2 1 2 1 1 2 2	0.0285
8	1 1 1 1 1 1 1 1 1 1 1 1 1 2 1 1 1 1 1 1	0.024
9	1 1 1 1 1 1 1 1 1 1 1 1 1 1 1 1 1 1 2 2	0.021
10	1 1 1 1 2 1 1 1 1 2 1 1 1 1 1 2 1 1 1 1	0.0205
11	1 1 2 1 1 1 1 1 1 1 1 1 1 1 1 1 1 1 2 1	0.0185
12	1 1 1 1 1 1 1 1 1 2 2 1 1 1 1 2 1 1 1 1	0.0165
13	1 1 2 2 1 1 1 1 1 1 1 1 2 1 1 2 1 2 2 2	0.0135
14	1 1 1 1 2 1 1 1 1 2 2 1 1 1 1 2 1 1 2 1	0.0105
15	1 1 2 1 1 2 2 2 2 1 1 1 2 1 1 2 1 1 1 1	0.01
16	1 1 1 1 1 1 1 1 1 2 2 2 2 2 1 2 1 1 1 2	0.0095
17	1 2 1 1 1 2 2 2 2 1 1 1 2 1 1 2 1 1 1 1	0.0095
18	1 1 1 1 2 1 1 1 1 2 2 1 1 1 1 2 2 2 1 1	0.009
19	1 1 1 1 1 1 1 1 1 2 2 1 1 1 1 2 1 1 2 2	0.0085
20	1 1 1 1 2 1 1 1 1 1 1 1 2 1 1 2 1 1 2 2	0.007
21	1 1 2 2 1 1 1 1 1 1 1 1 2 1 1 2 1 1 2 2	0.0065
22	1 2 1 1 1 2 2 2 2 1 1 1 2 1 1 2 2 2 2 1	0.006
23	2 1 2 2 1 1 1 1 1 2 2 1 1 1 1 2 1 1 1 1	0.0055
24	1 1 1 1 1 1 1 1 1 2 2 1 1 2 1 2 1 1 1 2	0.005
25	1 1 1 2 1 1 1 2 2 2 2 1 2 2 1 2 1 1 2 2	0.0045
26	1 2 1 1 1 2 2 2 2 1 1 1 2 1 1 2 1 1 2 1	0.0045
27	1 2 1 1 1 2 2 2 2 1 1 1 2 1 2 2 1 1 1 1	0.0045
28	1 1 1 2 1 1 1 2 2 2 2 1 2 2 1 2 1 1 1 1	0.0035
29	1 1 1 1 1 1 1 1 1 1 1 1 1 1 1 1 1 1 1 2	0.003
30	1 1 1 1 1 1 1 1 1 1 1 1 1 1 1 2 1 1 1 2	0.0025
31	2 1 2 2 1 1 1 1 1 2 2 2 2 2 1 2 1 1 1 1	0.002
32	1 1 1 1 1 1 1 1 1 1 1 1 1 1 1 2 1 1 2 2	0.0015
33	1 1 1 1 1 1 1 1 1 1 1 1 1 1 1 2 1 1 1 1	0.001
34	1 2 1 1 1 2 2 2 2 1 1 1 1 1 1 1 1 1 2 2	0.001
35	1 2 1 1 1 2 2 2 2 1 1 1 2 1 1 2 1 1 1 2	0.001
36	2 1 2 2 1 1 1 1 1 1 1 1 1 1 1 1 1 1 1 1	0.001
37	1 1 1 1 1 1 1 1 1 1 1 1 1 1 1 1 2 2 1 1	0.0005
38	1 1 1 1 1 1 1 1 1 1 1 1 1 1 1 2 1 1 2 1	0.0005
39	1 1 1 1 1 1 1 1 1 1 1 1 1 1 1 2 2 2 1 1	0.0005
40	1 1 1 1 1 1 1 1 1 1 1 1 1 1 1 2 2 2 2 1	0.0005
41	1 1 1 1 1 1 1 1 1 1 1 1 1 2 1 1 1 1 2 2	0.0005
42	1 1 1 1 1 1 1 1 1 1 1 1 1 2 1 2 1 1 1 2	0.0005
43	1 1 1 1 1 1 1 1 1 1 1 1 1 2 1 2 1 1 2 1	0.0005
44	1 1 1 1 1 1 1 1 1 1 1 1 2 1 1 2 2 2 1 1	0.0005
45	1 1 1 1 1 1 1 1 1 1 1 1 2 2 1 2 1 1 1 2	0.0005
46	1 1 1 1 1 1 1 1 1 1 1 1 2 2 1 2 1 1 2 1	0.0005
47	1 1 1 1 1 1 1 1 1 2 2 1 1 1 1 1 1 1 1 1	0.0005
48	1 1 1 1 1 1 1 1 1 2 2 1 1 2 1 2 1 1 2 2	0.0005

49	1 1 1 1 1 1 1 1 1 2 2 1 2 2 1 2 1 1 2 1	0.0005
50	1 1 1 1 1 1 1 2 2 1 1 1 2 1 1 2 2 2 1 1	0.0005
51	1 1 1 2 1 1 1 1 1 1 1 1 1 1 1 1 1 1 1 1	0.0005
52	1 1 1 2 1 1 1 1 1 2 2 2 2 2 1 2 1 1 1 1	0.0005
53	1 1 1 2 1 1 1 2 2 1 1 1 1 1 1 1 1 1 1 1	0.0005
54	1 1 1 2 1 1 1 2 2 2 2 1 2 2 1 2 1 1 1 2	0.0005
55	1 1 1 2 1 1 1 2 2 2 2 1 2 2 1 2 2 2 1 1	0.0005
56	1 1 2 1 1 1 1 1 1 1 1 1 1 1 1 1 1 1 2 2	0.0005
57	1 1 2 1 1 1 1 1 1 1 1 1 1 1 1 2 2 2 1 1	0.0005
58	1 1 2 1 1 2 2 2 2 1 1 1 1 1 1 1 1 1 1 1	0.0005
59	1 1 2 1 1 2 2 2 2 1 1 1 2 1 1 2 1 1 1 2	0.0005
60	1 1 2 1 1 2 2 2 2 1 1 1 2 1 1 2 1 1 2 1	0.0005
61	1 1 2 2 1 1 1 1 1 2 2 2 2 2 1 2 1 1 2 2	0.0005
62	1 2 1 1 1 1 1 1 1 1 1 1 1 1 1 1 1 1 1 1	0.0005
63	1 2 1 1 1 2 2 2 2 1 1 1 2 1 1 2 2 2 1 2	0.0005
64	2 1 1 1 1 1 1 1 1 1 1 1 1 1 1 1 1 1 1 1	0.0005
65	2 1 2 2 1 1 1 1 1 2 2 2 2 2 1 1 1 1 1 1	0.0005

Table A3 - Power of F_HapMiner and the single-locus TDT (SL-TDT) using GAW dataset on the single locus model (sample size = 50 pedigrees).

SNP	MAF	Penetrance Set A		Penetrance Set B		Penetrance Set C	
		SL-TDT	F_HapMiner	SL-TDT	F_HapMiner	SL-TDT	F_HapMiner
1	0.139	0.50	0.83	0.97	1.00	0.96	1.00
2	0.076	0.24	0.56	0.92	0.99	0.91	1.00
3	0.19	0.49	0.70	0.87	0.99	0.97	1.00
4	0.2295	0.64	0.89	0.93	0.99	0.99	1.00
5	0.047	0.07	0.20	0.20	0.43	0.24	0.31
6	0.087	0.34	0.56	0.96	1.00	0.97	1.00
7	0.087	0.38	0.67	0.98	1.00	0.96	1.00
8	0.1285	0.46	0.76	0.96	1.00	0.98	1.00
9	0.1285	0.48	0.73	0.95	1.00	0.96	1.00
10	0.2885	0.56	0.87	0.92	0.98	0.99	1.00
11	0.268	0.68	0.91	0.94	1.00	0.99	1.00
12	0.171	0.56	0.82	0.92	1.00	0.99	1.00
13	0.3265	0.62	0.90	0.80	0.98	0.98	1.00
14	0.244	0.61	0.82	0.78	0.99	0.97	1.00
15	0.0045	0.01	0.01	0.03	0.02	0.01	0.01
16	0.41	0.49	0.67	0.65	0.81	0.98	1.00
17	0.0675	0.28	0.49	0.93	1.00	0.84	0.97
18	0.081	0.21	0.59	0.89	0.98	0.79	0.99
19	0.289	0.08	0.19	0.14	0.32	0.20	0.32
20	0.247	0.58	0.73	0.77	0.93	0.96	1.00

Figure A1 - A CEPH pedigree

Typical CEPH pedigrees in our simulation have two or three generations with 4-20 members.

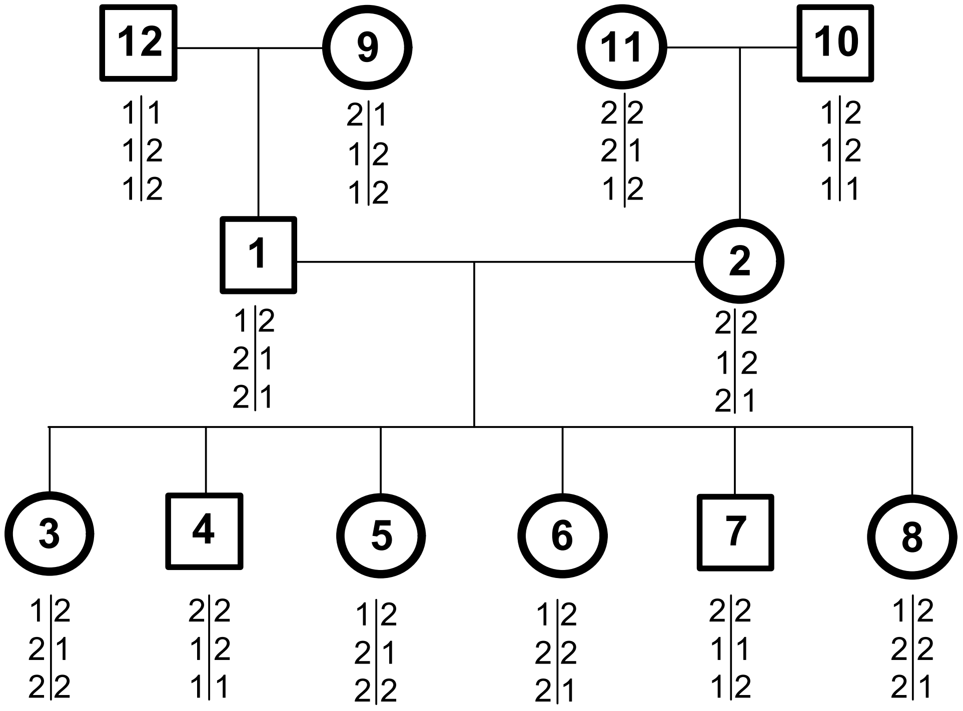

Figure A2 - The LD plot of the CF dataset

The block structure was generated based on haplotype frequencies of the CF data using the program of HaploView. It clearly shows that no nearby SNPs are in high LD with SNP 4.

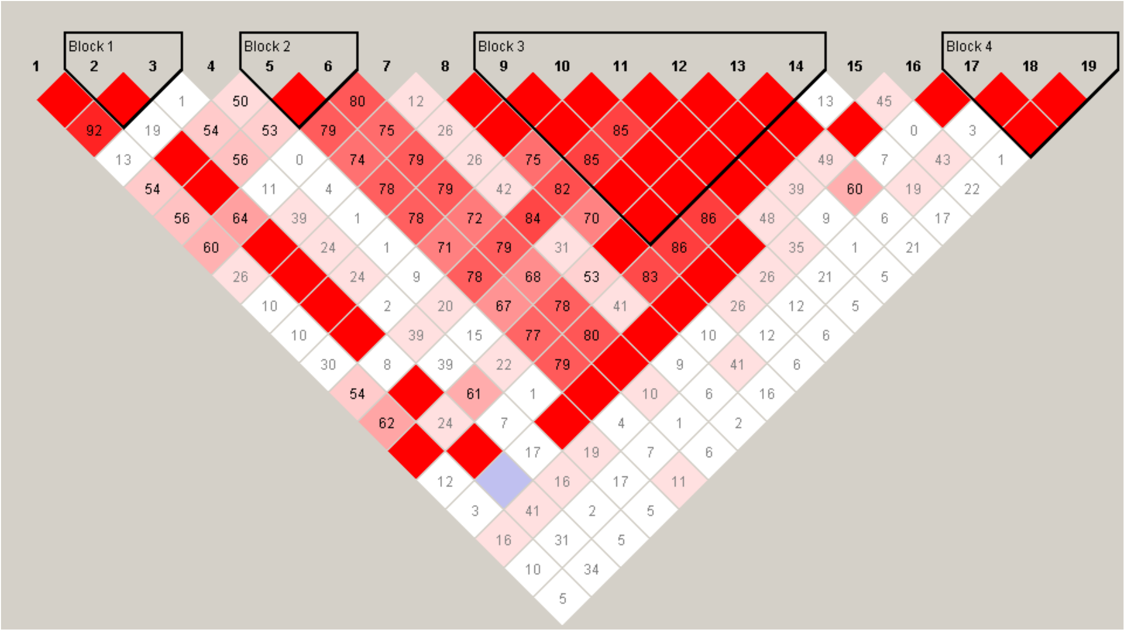

Figure A3 - The LD plot of the GAW dataset

The block structure was generated based on haplotype frequencies of the GAW data using HaploView. The block structure clearly shows that no SNPs are in high LD with SNP 19.

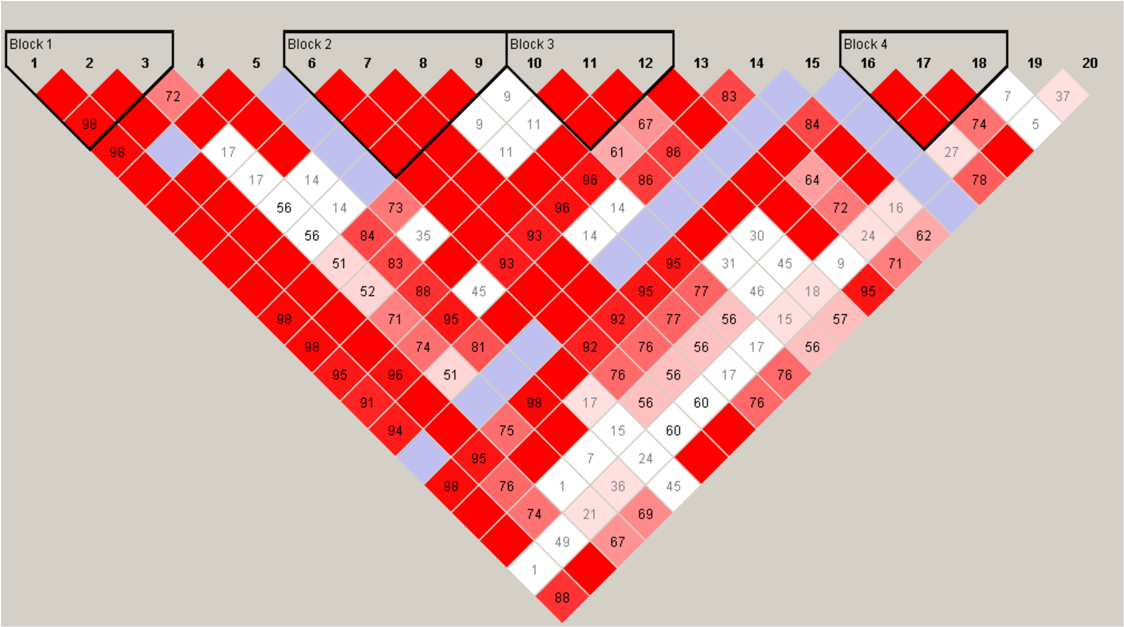

Figure A4 - Effect of Sample Sizes

Power of F_HapMiner increases when the sample size increases from 200, to 300, and 400. Result is based on the rare haplotype model with the penetrance set A.

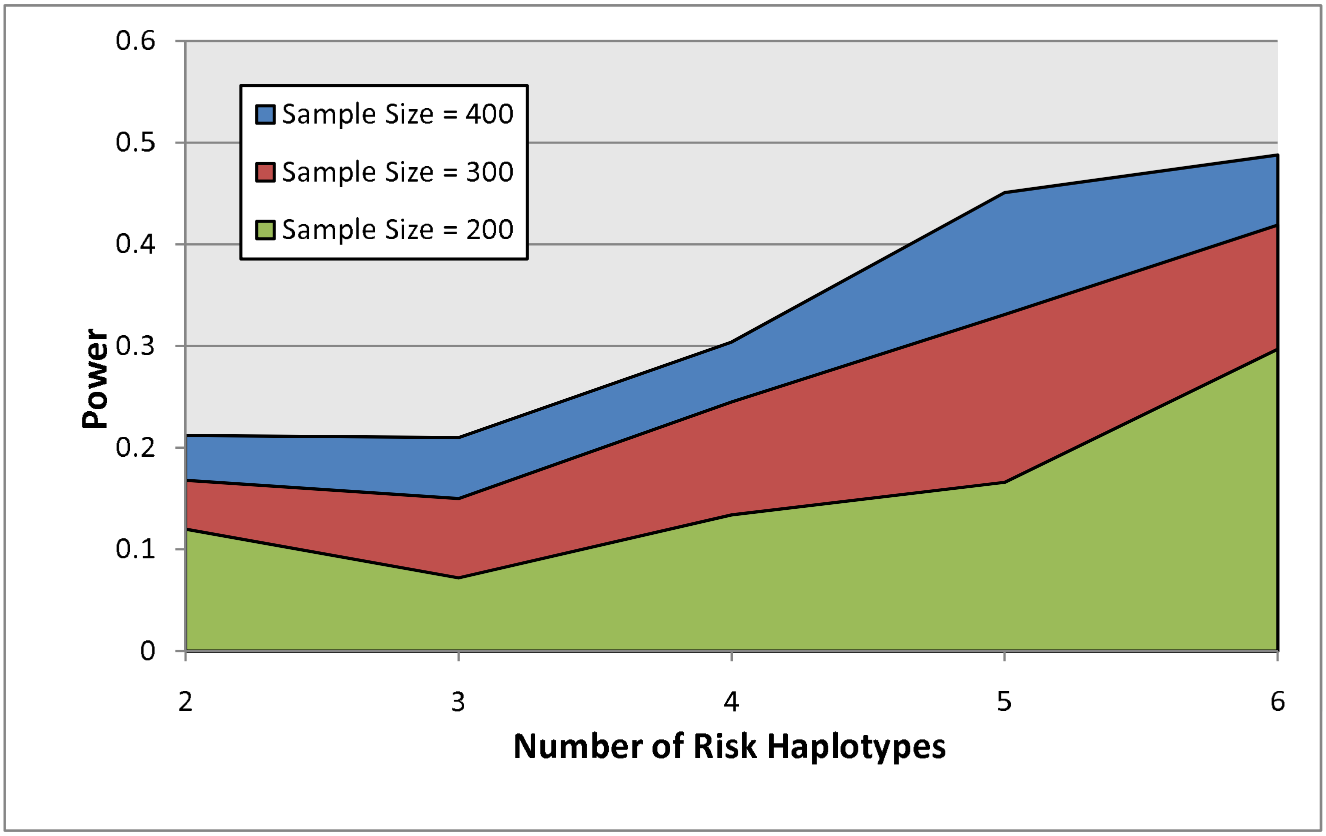

Supplement: Additional file 1 [file 1471-2105-11-S1-S45-S1.pdf]
